# Integrated mRNA and miRNA expression profile analysis of female and male gonads in *Hyriopsis cumingii*

**DOI:** 10.1038/s41598-020-80264-7

**Published:** 2021-01-12

**Authors:** Ya-Yu Wang, Sheng-Hua Duan, Gui-Ling Wang, Jia-Le Li

**Affiliations:** 1 Key Laboratory of Freshwater Aquatic Genetic Resources, Ministry of Agriculture and Rural Affairs, Shanghai Ocean University, 999 Huchenghuan Road, Shanghai, 201306 China; 2 National Demonstration Center for Experimental Fisheries Science Education, Shanghai, 201306 China; 3 Shanghai Engineering Research Center of Aquaculture, Shanghai, 201306 China

**Keywords:** RNA sequencing, Sequencing

## Abstract

*Hyriopsis cumingii* is an important species for freshwater pearl cultivation in China. In terms of pearl production, males have larger pearls and better glossiness than females, but there are few reports focusing on the sex of *H. cumingii*. In this study, six mRNA and six microRNA (miRNA) libraries were prepared from ovaries and testes. Additionally, 28,502 differentially expressed genes (DEGs) and 32 differentially expressed miRNAs (DEMs) were identified. Compared with testis, 14,360 mRNAs and 20 miRNAs were up-regulated in ovary, 14,142 mRNAs and 12 miRNAs were down-regulated. In DEGs, the known genes related to sex determinism and/or differentiation were also identified, such as *DMRT1*, *SOX9*, *SF1* for males, *FOXL2* for females, and other potentially significant candidate genes. Three sex-related pathways have also been identified, which are Wnt, Notch, and TGF-beta. In 32 DEMs, the three miRNAs (miR-9-5p, miR-92, miR-184) were paid more attention, they predicted 28 target genes, which may also be candidates for sex-related miRNAs and genes. Differential miRNAs target genes analysis reveals the pathway associated with oocyte meiosis and spermatogenesis. Overall, the findings of the study provide significant insights to enhance our understanding of sex differentiation and/or sex determination mechanisms for *H. cumingii*.

## Introduction

*Hyriopsis cumingii*, a kind of bivalve mollusk, its pearl is known by the smooth appearance and bright color^1^. It is a crucial freshwater pearl species with significantly economic in China. Studies have shown that the pearl rearing performance of *H. cumingii* is different between 3 and 4 years old females and males, such as the total weight, grain weight, and particle size of pearls. The average pearl weight of male mussels is 12.4–17.5% higher than that of females. Besides, The average grain weight is 13.5–17.9% higher, and the average grain size is 4.4–5.4% higher^2^. Obviously, the male *H. cumingii* has better pearl breeding performance. Hence, sex is an important factor affecting the yield and quality of the pearl. However, the present studies on sex determination or sex differentiation of bivalve shellfish are very few, which restricts the development of the pearl industry. Therefore, it is indispensable to understand the mechanism of sex determination and/or differentiation in *H. cumingii*.

To date, there are relatively few researches on the genetic and phenotypic basis of bivalve sex differentiation, especially in the molecular pathway of reproduction. Their reproductive system is different by the hermaphroditic, dioecism, and gender changes. For instance, as we well-known *Crassostrea gigas*, it is an alternative and irregular protandrous hermaphrodite: most individuals mature first as males and then change sex several times^3^. Some species are hermaphroditic. For example, *Nodipecten subnodosus* is a functional hermaphrodite in which male and female gametes mature at the same time^4^. Additionally, species like *Placopecten magellanicus* are dioecious^5^. In recent years, the development of high-throughput sequencing technology has accelerated the identification of transcripts. Studies on gonadal development, sex differentiation and sex determination of bivalve shellfish are increasingly in-depth at the same time. The transcriptome analysis of *N. subnodosus* illustrated that the genes of the sex determination/differentiation are *Ns-SEX1*, *Ns-DMRTA2*, *Ns-SOX9*, *Ns-WNT4*, *Ns-DOA*, *Ns-OVO*, and *Ns-VIR*^4^. In the differential gene analysis of the gonadal transcript of blood clam (*Tegillarca granosa*), sex-related genes included *FOXL2*, *SOX*, *β-CATENIN*, *CBX*, and *SXL*^6^. In yesso scallop (*Patinopecten yessoensis*), a hypothetical sex determination and differentiation pathway was constructed, in which *PyDMRT1* may have a leading function^7^. The gonadal transcriptome of *Pinctada Margaritifera* revealed the importance of *pmarg-FOXL2* and *pmarg-FEM1-like* for sex inversion and sex differentiation, which provided a powerful resource for the molecular mechanism of reproductive strategy in hermaphroditic mollusks^8^. These results may suggest that genes such as *FOXL2*, *DMRT*, *SOX* can play a role during sex regulation in many species, indicating that their functions are very conservative. MicroRNA is a single-stranded non-coding RNA molecule with a length of about 21–24 nucleotides. And it is a post-transcriptional regulator. miRNAs sequencing has advanced our understanding of miRNAs in biological sex differentiation and gonadal development, and it has been applied in a variety of aquatic species. The sex-biased miRNAs (such as miR-9, miR-21, miR-30a, miR-96, miR-200b, miR-212 and miR-7977) were obtained by a comprehensive analysis of miRNA and mRNA expression profiles in the gonads of tilapia at the early stage of sexual differentiation. Their target genes include *FOXL2*, *AMH*, *STAR1*, *SF1* and *DMRT1*, which are key molecules involved in vertebrate sex differentiation^9^. Three significant miRNAs (aca-miR-30b-5p, ame-miR-263b and cfa-miR-125a) were screened from *Macrobrachium nipponense*. They and their predicted target genes may have a strong impact on sex differentiation/determination^10^. miRNAs were also identified and analyzed during the gonadal development of *Macrobrachium rosenbergii* and zebrafish^11,12^. These data will be useful for further study of miRNA mediated gonadal development and reproductive regulation mechanism. In animals, miRNAs regulate target gene expression through degradation or translation inhibition^13^. The relationship between miRNA and mRNA is not a one-to-one correspondence. A single miRNA can target multiple mRNAs, and one mRNA can also have several miRNAs binding sites^14^. During gonadal development, miRNAs are expressed differently in male and female gonads. They play a role through germ cells and gonadal cells to regulate critical proteins needed for gonadal development^15^. Studies have shown that miR-202-5p/3p can be expressed in a sexual dimorphism pattern as the primordial XY gonad differentiates into a testis and play an early role in mouse testis development^16^. The results of knockdown or overexpression of miR-124 in mouse showed that miR-124 could induce the repression of both *SOX9* translation and transcription in ovarian cells^17^. Therefore, understanding the regulatory relationship between miRNA and sex-determining genes can help us have a better comprehending of the sex-determining mechanism.

Bioinformatics analysis of miRNA and mRNA expression profile can help us improve the reliability of prediction and understand the molecular mechanism of post-transcriptional regulation. Nowadays, there is no information about the interaction network and regulation of mRNAs and miRNAs in the gonads of *H. cumingii*. Therefore, our goal is to identify the essential genes and miRNAs that regulate sex determination/differentiation of *H. cumingii* by mRNA sequencing and miRNA sequencing. This study provides basic information for understanding sex differentiation and sex determining mRNAs and miRNAs of *H. cumingii*.

## Results

### mRNA sequencing and assembly

The samples were sequenced from the gonads of six *H. cumingii*, including three ovaries (F1, F2, and F3) and three testes (M1, M2, and M3). A total of 308,008,006 raw reads were produced by Illumina HiSeq X Ten sequencing. And 302,865,864 clean reads filtered by Trimomatic. The average percentage of Q30 and the G + C percentage of the six cDNA libraries were 94.75% and 41.48% (Table 1). After De novo assembly, the number of unigenes is 96,266. The length range is 301–28,485 bp. And the average length is 1101.67 bp. The annotated results of the genes in seven databases are NR 21,790 (22.64%), Swissprot 14,705 (15.28%), KEGG 9573 (9.94%), KOG 11,833 (12.29%), eggNOG 16,525 (17.17%), GO 13,429 (13.95%), Pfam 14,987 (15.57%).

**Table 1:** Summary information and analysis of mRNA sequences in *H. cumingii.*

Sample	Raw reads	Clean reads	Q30 (%)	GC (%)
F1	53,449,060	52,542,528	94.67	40.89
F2	49,936,082	49,120,634	94.96	40.77
F3	50,929,990	50,033,610	94.83	40.67
M1	46,700,762	45,946,032	94.70	42.41
M2	53,239,136	52,374,040	94.84	43.12
M3	53,752,976	52,849,020	94.50	41.04

Raw reads: the statistics of the raw reads; Clean reads: remove the contaminated and low quality read; Q30: The ratio of bases with a value greater than 30 to the total bases in raw bases; GC: The ratio of the total number of G and C to the total number of bases in clean bases.

### Screening for DEGs and functional annotation

Three female gonadal tissues (F1 ~ F3) were Group_F, and three male gonadal tissues (M1 ~ M3) were Group_M. Compared with Group_M, there were 28,502 DEGs in Group_F, including 14,360 genes up-regulated and 14,142 genes down-regulated. GO annotations consist of three categories: biological processes, cellular components, and molecular functions. DEGs were enriched to 9295 GO terms. Ten terms with *P* value less than 0.05 and the largest number of DEGs were selected in each of the three categories (Supplementary Fig. 1). KEGG enrichment of DEGs showed that there were 305 pathways, remove the pathways in which the number of DEGs is less than three, sort them according to the − log_10_ *P* value, and draw a bubble diagram with the first 20 pathways (Supplementary Fig. 2).

According to sex determining genes of other species^18,19^ and GO analysis, we screened 12 sex-related genes in *H. cumingii* transcriptomes (Table 2), including *DMRT*, *TRA*, *SOX*, *FOXL* genes, etc. GO analysis is to find candidate genes by searching for "sex" keywords in GO terms. The search results include nine GO terms, namely sex determination (GO:0,007,530), sex differentiation (GO:0,007,548), male sex differentiation (GO:0,046,661), male sex determination (GO:0,030,238), female somatic sex determination (GO:0,019,101), sex determination establishment of X:A ratio (GO:0,007,540), sex chromosome (GO:0,000,803) , male germ-line sex determination (GO:0,019,100), and development of primary female sexual characteristics (GO:0,046,545). The DEGs in these terms are listed in Table 2. In the KEGG enrichment results, Wnt (ko04310), Notch (ko04330), and TGF-beta (ko04350) were confirmed to be related to sex determination^20^. There are 49 DEGs in these three signaling pathways, which may be related to sex regulation of *H. cumingii* (Supplementary Table 1). Through these methods, we obtained a total of 67 sex-related candidate genes (after removing duplicates). 14 differentially expressed genes were selected for quantitative real-time PCR (qRT-PCR) detection (Supplementary Fig. 3).

**Table 2:** List of important genes related to sex determination/differentiation in the *H. cumingii* transcriptomes.

Gene	NR id	Name
*SOX9*	AGI96396.1	SRY-related HMG-domain containing transcription factor 9
*DMRT1*	ASV71764.1	Double-sex and mab-3 related transcription factor 1
*FOXL2*	XP_022345405.1	Forkhead box protein L2
*SF1*	XP_011417019.1	Splicing factor 1
*TRA2*	XP_025096128.1 XP_021378792.1	Transformer-2
*BMP2*	OWF43831.1	Bone morphogenetic protein 2
*ZGLP1*	XP_011445224.2	GATA-type zinc finger protein 1
*CYP17A1*	XP_025084033.1 XP_021361085.1	Steroid 17-alpha-hydroxylase/17,20 lyase
*DACH2*	XP_021340457.1	Dachshund homolog 2
*DPN*	AGS55441.1	Hairy enhancer of split 7
*SMC5*	XP_021362293.1	Structural maintenance of chromosomes protein 5
*SRD5A1*	EKC36980.1	3-oxo-5-alpha-steroid 4-dehydrogenase 1

### miRNAs sequencing and assembly

We constructed six cDNA libraries of small RNAs from F1, F2, F3, M1, M2, and M3. After filtering the Rfam database, transcript sequence, and Repbase database, the reads are compared with miRBase to identify and annotate the known miRNA (Table 3). The number of miRNAs in all samples was 209, including 84 known miRNAs and 125 novel miRNAs.

**Table 3:** Summary information and analysis of miRNA sequences in *H. cumingii.*

Sample	Raw reads	Clean reads	Reads trimmed Q20	Known miRNA	Novel miRNA
F1	30,286,657	18,147,307	18,163,822	65	81
F2	41,577,946	29,989,345	30,009,397	65	93
F3	36,741,132	19,784,177	19,811,678	66	92
M1	37,370,743	28,974,936	28,988,411	66	88
M2	29,870,390	18,486,304	18,500,540	70	74
M3	37,527,689	26,717,472	26,755,846	74	85

Raw reads: the statistics of the raw reads; Clean reads: remove the contaminated and low quality read; Reads trimmed Q20: reserve the reads of the percent of Q20 > 80%.

**Figure 1:**
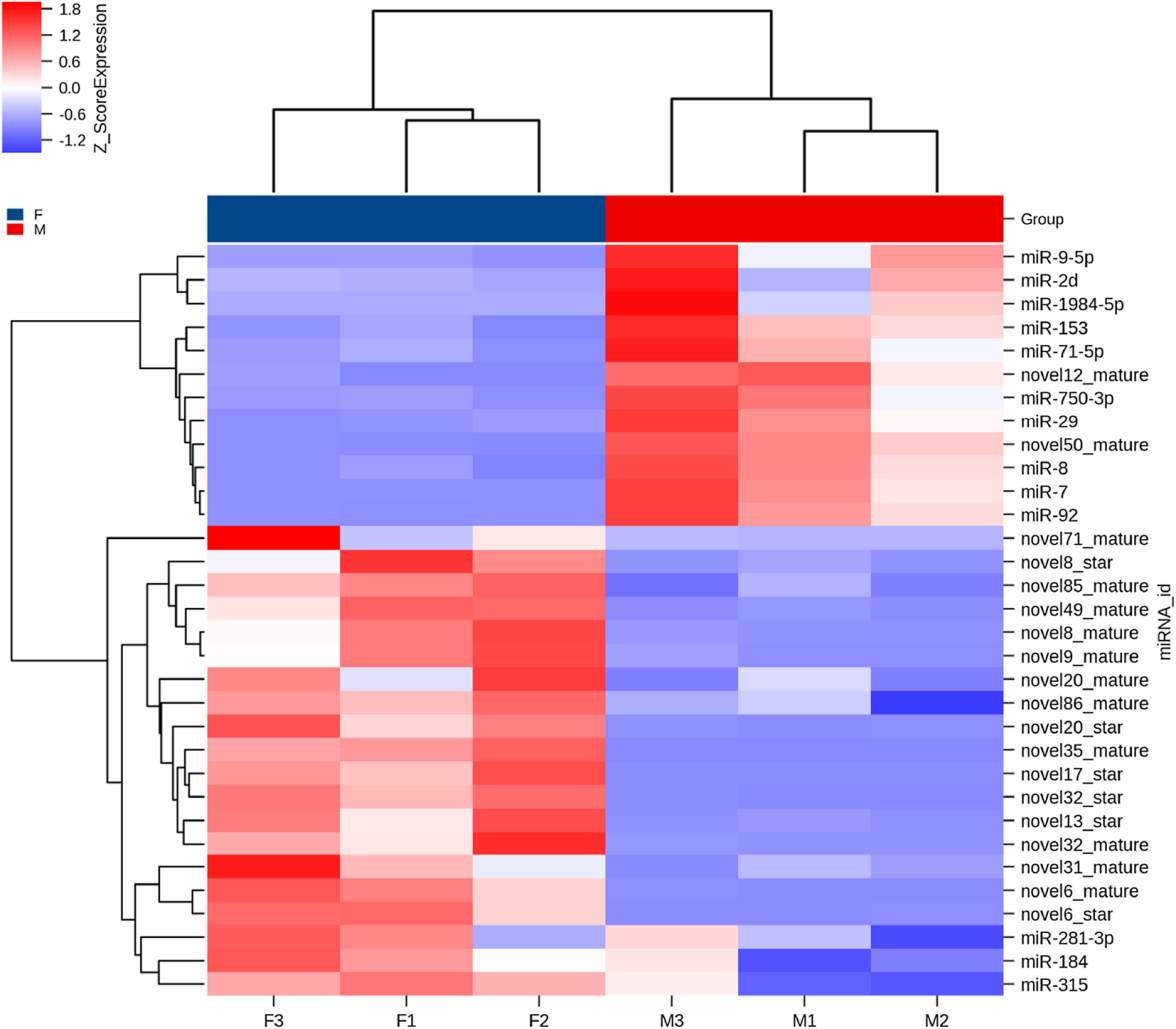
Heatmap plots for differentially expressed miRNAs between Group_F and Group_M. Sample names are represented in columns and significant miRNAs are represented in rows. miRNAs are clustered together based on their expression similarity. The color indicates the Z_score from high (red) to low (blue).

**Figure 2:**
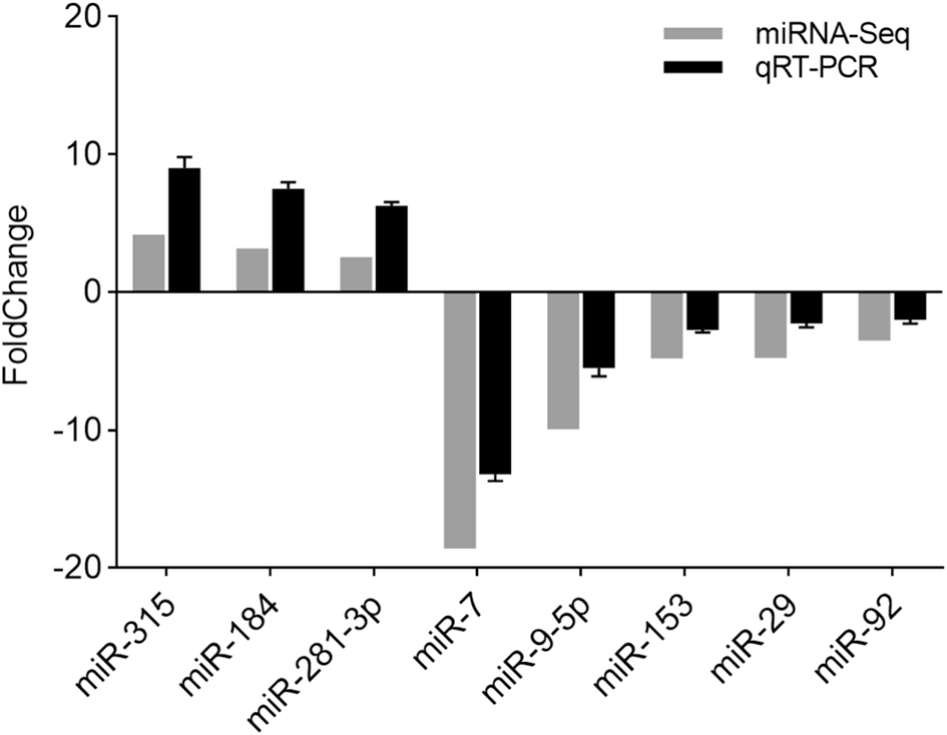
The relative expression of different miRNAs revealed by real-time quantitative PCR. Data are shown as mean ± SD (standard deviation) of tissues from three separate individuals. The figure is drawn by GraphPad Prism (version 8.0.1) software (https://www.graphpad.com/).

### Screening for Differentially-expressed miRNAs (DEMs) and functional annotation

Compare with miRNA libraries of Group_F vs. Group_M, 32 DEMs were obtained, including 13 known miRNAs and 19 novel miRNAs (Supplementary Table 2). Among the known miRNAs, three were ovary-biased, and ten were testis-biased. Unsupervised hierarchical clustering groups the samples in two major clusters (Fig. 1). The number of target genes of 32 DEMs are 911. The functional terms cytoplasm, ATP binding, and nucleoplasm, had a higher number of transcripts than other terms (Supplementary Fig. 4). KEGG analysis showed that the target genes were enriched in 184 signaling pathways. The largest number of target genes is found in cell cycle (Supplementary Fig. 5). To study the expression pattern of these DEMs in testis and ovary, we randomly selected three ovary-biased miRNAs (miR-184, miR-281-3p, miR-315), and five testis-biased miRNAs (miR-153, miR-29, miR-7, miR-9-5p, miR-92) to verify their expression profiles by qRT-PCR (Fig. 2). All of these miRNAs expression patterns are consistent with the results of miRNAs sequencing, indicating that the sequencing results are reliable.

### Integrated analysis of DEMs and DEGs

**Figure 3:**
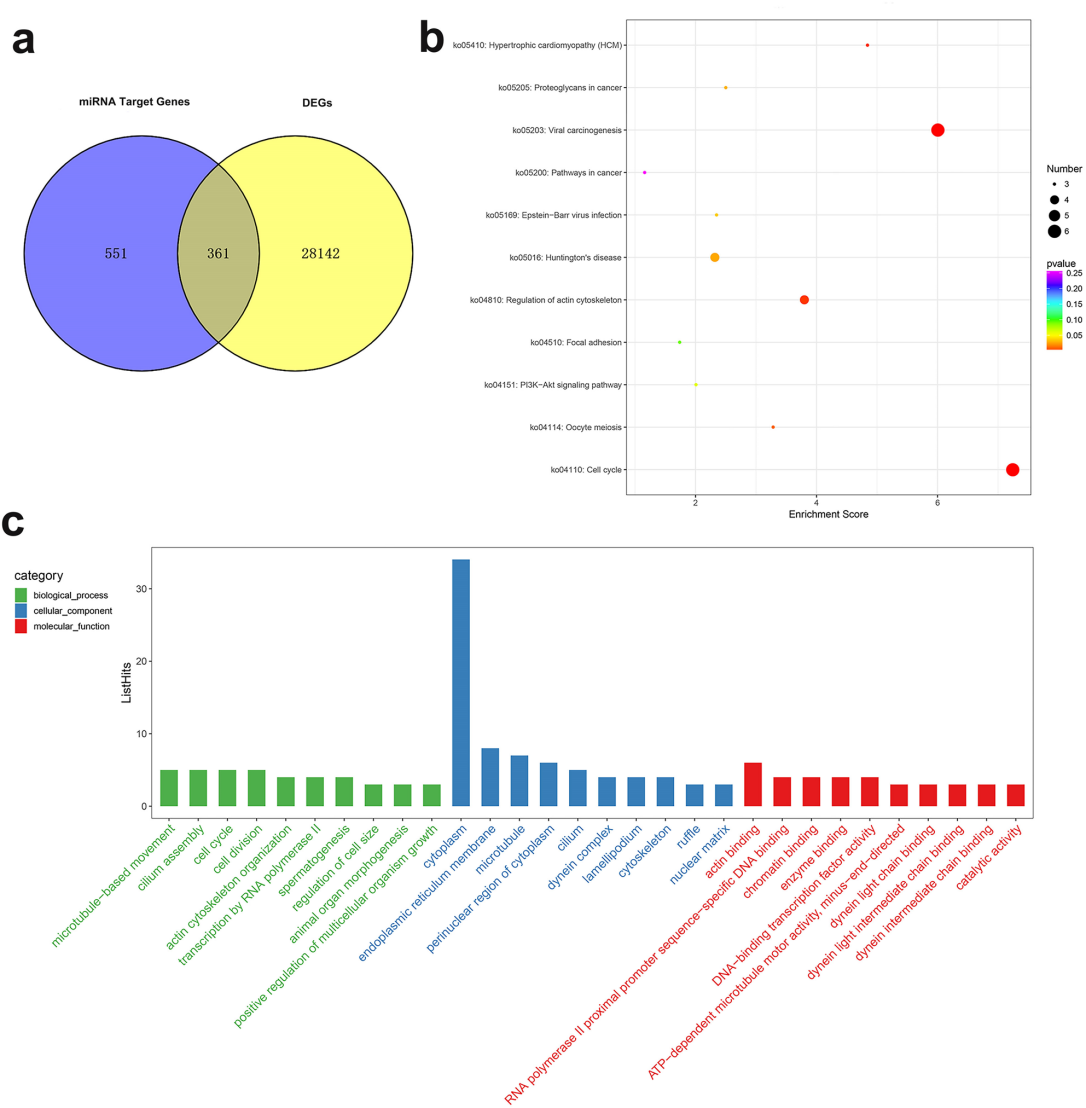
GO and KEGG analysis of differential target genes. (**a**) Integrate analysis of mRNA and miRNA. Venn diagram representing the intersection between differentially expressed genes (DEGs) and target genes of the differentially expressed miRNAs (DEMs) in the Group_F and Group_M. (**b**) Bubble diagramv of KEGG enrichment analysis. (**c**) GO enrichment analysis.

According to the results of transcriptome sequencing and miRNAs sequencing, we found three key miRNAs (miR-9-5p, miR-92, miR-184) and their target genes (Table 4). These genes may be related to the sex regulation of *H. cumingii*. Some of them have been reported to relates to sex. For example, *REX1* is associated with X-chromosome inactivation in mice^21^. *SMAD4* is essential factors that regulate the female fate of human germ cells^22^. *MCM6* was identified and considered as candidate genes that may be involved in sex differentiation regulation in cucumber^23^. *CDH5* is necessary for spermatogenesis and chromatin concentration in mice^24^. Besides, 361 differentially-expressed target genes were obtained from the intersection of 28,502 DEGs and 911 target genes (Fig. 3a). GO analysis showed that there were more transcripts in the cytoplasm, ATP binding, calcium ion binding, endoplasmic reticulum membrane, and microtubule (Fig. 3c). In the biological process, there is a term for spermatogenesis (GO:0,007,283), in which the genes are *IFT81*, *LRGUK*, *SYNE1*, *TDRD1*. KEGG analysis showed that 361 differentially-expressed target genes were mapped to 113 pathways. There are many genes involved in the pathways of cell cycle, viral carcinogenesis, and regulation of actin cytoskeleton (Fig. 3b). And there is an oocyte meiosis signal pathway (ko04114), which contained three genes: *PPP1C*, *PKMYT*, and *CCNE*. These genes were randomly selected for qRT-PCR verification, *SYNE1* and *TDRD1* were highly expressed in the ovary, and *IFT81*, *PPP1C*, *PKMYT*, and *LRGUK* were highly expressed in testis (Supplementary Fig. 3). The corresponding miRNAs of the seven genes (*IFT81*, *LRGUK*, *SYNE1*, *TDRD1*, *PPP1C*, *PKMYT*, and *CCNE*) related to spermatogenesis and oocyte meiosis are novel31, novel85, miR-71-5p, miR-750-3p, novel31, novel85, novel86, respectively.

**Table 4:** The predicted target genes of miR-9-5p, miR-92 and miR-184.

miRNA	Targeted genes	NR id
miR-9-5p	*BLM*, *GATD3A*, *REX1*, *CHIA*	OWF37785.1, XP_013391615.1, OWF37872.1, XP_012945342.1
miR-92	*RDH16*, *HEXA_B*, *SMAD4*, *LRP4*, *HM20A*, *MCM6*, *PNKP*, *ADAMTS18*, *CG9801*, *MOB1*, *MUT*, *MARK1*	XP_014596438.1, XP_015772465.1, XP_021379469.1, XP_019918967.1, XP_011423614.1, XP_021360010.1, XP_022344800.1, OWF36531.1, OWF39070.1, XP_021349454.1, XP_019920763.1, XP_021360442.1
miR-184	*SVEP1*, *ZASP52*, *LRCH3*, *CHD5*, *SESN1*, *BCKDK*, *POL*, *ATP6V1FNB*, *MUS81*, *TEAD*, *LRCH3*, *PAXBP1*	XP_021344288.1, KOF67579.1, XP_021371746.1, XP_025081588.1, XP_021352570.1, XP_014784793.1, XP_021346951.1, OWF34682.1, XP_013385301.1, XP_013063509.1, XP_021371746.1, OWF45544.1

## Discussion

In order to explore the mRNAs and miRNAs involved in sex differentiation and/or sex determination of *H. cumingii*, RNA-seq and miRNA-seq analyses were performed on ovaries and testes. To the best of our knowledge, this is the first report of miRNA and mRNA profiling of ovary and testis in *H. cumingii*. We analyzed miRNAs and mRNAs to provide a basis for screening candidate miRNAs and sex-related genes. This result provides an essential insight into the mechanism of sex differentiation/determination in *H. cumingii*.

Since there are few studies focusing on the sex of bivalve shellfish, to find candidate genes related to sex determination, we refer to other species genes that have been identified, which has important reference value for our research. After screening the transcriptome results, we obtained seven DEGs as *SOX9*, *DMRT1*, *FOXL2*, *SF1*, *TRA2*, *BMP2* and *ZGLP1*. In general, *SOX9* is a direct target of *SRY* gene in mammals and is necessary for normal testicular development^25^. The conservative expression of *SOX9* in chicken and mouse indicates its primary role in the testicular determination of vertebrates^26^. *SOX9* was also identified in bivalve shellfish, such as *N. subnodosus*, *C. gigas*, and *Hyriopsis schlegelii*^4,27,28^. In our results, miranda software predicted that there was an interaction site between miR-193 and *SOX9*. Also, *SOX9* was highly expressed in testis (Supplementary Fig. 3). These results suggest the potential role of *SOX9* and miR-193 in the sex of *H. cumingii*. Unfortunately, due to the limitation of miRNA sequencing information, other genes were not predicted by miRNAs except *SOX9*. Apart from that, *DMRT* (double sex/male-normal-3-related transcription factor) gene is a major transcription factor controlling sex determination and differentiation^29^. *DMRT1* can not only control testicular differentiation and meiosis of male germ cells in mice^30,31^. But also be necessary for male sexual development in zebrafish^32^. In mollusks, the homologous gene of *DMRT* has been identified in many species, such as *C. gigas*, *P. margaritifera*, and *P. yessoensis*^33–35^. In *H. cumingii*, the qRT-PCR results showed that *DMRT1* gene was a testis specific gene, and its expression level in the ovary was very low, and the expression level in mRNA sequencing results was not detected (Supplementary Fig. 3). It was suggested that *DMRT1* was crucial for the male sexual development of *H. cumingii*. *FOXL2*, a member of the forkhead box (Fox) domain transcription factor family, plays a key role in ovarian differentiation and oogenesis in vertebrates^36^. *FOXL2* can affect ovarian development and sex determination of mice^37^. In *C. farreri* and *P. yessoensis*, *FOXL2* expression was a sexually dimorphic pattern, and the expression level in ovary was significantly higher than that in testis^38,39^. In our results, *FOXL2* was highly expressed in the ovary, and the expression abundance in the testis was very low. The results of mRNA sequencing also showed that *FOXL2* was not expressed in testis (Supplementary Fig. 3). It is suggested that *FOXL2* is crucial for the female sexual development of *H. cumingii*. *SF1* (Steroidogenic factor 1) is an important transcription factor involved in steroidogenesis, reproduction, and sexual differentiation^40^. The *TRA2* gene, encoding a protein (TRA-2) which directs sex-specifically alternative splicing, has been proved to play important roles in sex differentiation and sex development of Drosophila melanogaster^41^. *BMP2* (Bone morphogenetic protein 2) has been confirmed to be related to the fetal ovarian development and gonadal somatic cell differentiation of mice^42,43^. *ZGLP1* (GATA-type zinc finger protein 1) is essential for the oogenic program and meiotic entry in mice^44^. In addition to the above genes, GO analysis also enriched some extra sex determination/differentiation genes, they are *CYP17A1*, *DACH2*, *DPN*, *SMC5*, and *SRD5A1*. *CYP17A1* has the activity of 17α-hydroxylase and 17, 20-lyase, which is involved in the steroidogenic pathway that produces androgens and estrogens, it is crucial for male determination in amphibians^45^. *DACH* is differentially expressed in the male and female genital discs of drosophila, and plays sex-specific roles in the developmenting genitalia^46^. And *DPN* is also a primary part of sex determining signal of drosophila^47^. *SMC5* was confirmed to be associated with spermatogenesis of mice^48^. In *Crassostrea hongkongensis*, *SRD5A1* was expressed at a higher level in female gonads than in other tissues^18^. There is no doubt that the above role in gender regulation is vital. In the sequencing results, *FOXL2*, *BMP2*, *ZGLP1*, *CYP17A1*, *DACH2*, *DPN*, *SRD5A1* were highly expressed in the ovary, and *DMRT1*, *SF1*, *SMC5*, *SOX9* were highly expressed in the testis. This suggests that they may play a role in the formation of female or male. The results of random verification of qRT-PCR showed the accuracy of sequencing (Supplementary Fig. 3), but their position in sex regulation needs more research to explore. In KEGG results, the function of Wnt, Notch, and TGF-beta signaling pathways in sex determination and gonadal development in mammals have been relatively straightforward, including BMPs (bone morphogenetic proteins), HH (hedgehog), Hippo, nuclear receptors^20,49^. In addition to non-mammals, it has also been shown that the classical Wnt signaling pathway regulates gonadal differentiation in zebrafish^50^. The Notch signaling pathway can regulate stem cell proliferation and sex determination in the germline of *Caenorhabditis elegans*^51^. The role of *AMH* (anti-Müllerian hormone) in testicular differentiation of *Eriocheir sinensis* highlights the importance of TGF-beta pathway in sex determination of reptiles^52^. All three pathways exist in our results, indicating that they may be related to the sex determination/differentiation of *H. cumingii*. At the same time, in these three signal pathways, some gender-related genes have also been studied. *WNT2*, a wnt family ligand, has been demonstrated to be involved in the induction of sex-specific differences in drosophila^53^. *HES1* is an important transcription factor in Notch signaling pathway, which is related to the mouse testis cell fate determination step from primordial stage to the differentiated stage in adulthood^54^. The loss of *CREBBP* (*CREB* binding protein, Notch and TGF-beta signaling pathway member) and *P300* (its related paralogue) disrupts histone acetylation of mouse *SRY* promoter and causes XY gonadal sex reversal^55^. *CUL-1* (*CULLIN-1*, TGF-beta signaling pathway member), *SKR-1*, and *SEL-10* constitute a SCF E3 ligase complex that plays an critical role in modulating sex-determination of *C. elegans*^56^.

In this study, 32 miRNAs were identified from six miRNA libraries, among which 19 were novel miRNAs. Since novel miRNAs have not been studied in other species, we focus on 13 known miRNAs. The DEMs in our gonadal miRNA-seq results are similar to those of other species. For example, miR-7, miR-9, miR-29, miR-184 has been detected in miRNA-seq identification of other species^57–59^. In these miRNAs, miR-7 is a crucial factor in regulating the hypothalamus-pituitary-ovary axis of mice^60^. Moreover, miR-7a2 can regulate sexual maturation and reproductive function^61^. miR-9 is involved in spermatogenesis of spermatogonia during the natural sex change of *Monopterus albus*^62^*.* Furthermore, it can also regulate the critical genes in the ovarian development pathway of Mud Crab *Scylla paramamosain*^63^. miR-29 is an essential regulatory factor in the process of male meiosis of mice^64^. miR-184 was highly expressed in mouse testis, and down-regulated the expression of *NCOR2* during spermatogenesis^65^. In drosophila, miR-184 can affect oogenesis, early embryonic development, and ovarian development^66,67^. *DROSHA* is essential for the coordinated development of somatic and germ cell precursors in drosophila larvae. And miR-8 and miR-184 are regulated by *DROSHA*^67^. miR-92 may play a role in early chicken gonadogenesis^68^. In Japanese flounder, miR-92 regulates early development and metamorphosis of *Paralichthys olivaceus*^69^. We list three miRNAs and their target genes, and there are 28 DEGs (Table 4). Based on the potential function of these miRNAs in sex, it is speculated that these DEGs may also be sex-related candidate genes of *H. cumingii*. However, the specific functionality needs further experimental verification. Also, qRT-PCR results showed that miR-7, miR-9-5p, miR-29, miR-92, and miR-153 were highly expressed in the testis and miR-184, miR-218, miR-315 were highly expressed in the ovary (Fig. 2). This is consistent with our sequencing results, which further indicates that these DEMs may play a specific role in the sex regulation of *H. cumingii*.

361 differentially-expressed target genes were obtained from the intersection analysis of 28,502 DEGs genes and 911 target genes (Fig. 3a). These genes are differentially expressed in ovaries and testes, and their associated miRNAs are also differentially expressed between the two genders. There are two terms of spermatogenesis and oocyte meiosis in GO and KEGG analysis (Fig. 3b,c). They have seven differentially expressed genes (*IFT81*, *LRGUK*, *SYNE1*, *TDRD1*, *PPP1C*, *PKMYT*, and *CYCE*). In mouse spermatogenesis, *IFT81* plays an essential role in regulating the assembly and elongation of sperm flagellum^70^. And *LRGUK*-1 is required for basal body and manchette function during spermatogenesis and male fertility^71^. *PPP1C* is needed in the meiosis of mouse oocytes^72^. In *P. olivaceus*, *PoTDRD1* is a germ line specific and sex dimorphic factor, which may play a role in the development of its reproductive system and gametogenesis^73^. In *Xenopus*, *CPEB* and miR-15/16 co-regulate *CYCE1* and play a role in oocyte maturation^74^. These genes and miRNAs may be closely related to germ cell maturation and gametogenesis of *H. cumingii*.

## Conclusion

In this study, the transcriptome and miRNA analysis were applied to explore candidate genes/miRNAs for sex determination/differentiation in *H. cumingii*. mRNA and miRNA expression profiles provided a rich list of genes and miRNAs expressed in testes and ovaries. In total, 95 DEGs, three DEMs, and three signaling pathways were screened, which may be closely relative with sex determination/differentiation in *H. cumingii*. In the intersection analysis of DEGs and DEMs target genes, two signal pathways related to gametogenesis were obtained. They include seven genes and seven miRNAs. These sex-related genes, miRNAs, and signaling pathways provided not only the basis foundation for the study of the sexual regulation mechanism in *H. cumingii*, but also a fundamental reference for better understanding the sex determination and/or differentiation of bivalves.

## Materials and methods

### Ethics statement

The samples were collected from Jinhua farm in Zhejiang province. All experimental protocols were approved by the Institutional Animal Care and Use Committee (IACUC) of Shanghai Ocean University, Shanghai, China.

### Sample collection

Specimens used in current study were obtained WeiMing aquaculture farms (ZheJiang, China). Three sexually mature females and three males *H. cumingii* weighing 320–338 g were collected. In general, the mussels over 2 years old are considered to be sexually mature. The samples we used in this study were 3 years old. Because the eggs of *H. cumingii* are very large and the sperm is very small. We took a small amount of liquid from the gonads and observed them under a microscope to judge their sex. These mussels are propagated from the same batch of parents and cultured in the same culture pond. The water temperature in the pond ranges from 12 to 32 °C throughout the year, and the pH is about 7.5. All the samples were taken back to the laboratory of Shanghai Ocean University. Keep the mussels in a tank for 3 days. The water temperature was controlled around 26 °C. The gonadal tissues of three female mussels (F1, F2, F3) were Group_F, and those of three male mussels (M1, M2, M3) were Group_M. Gonadal samples were collected with high temperature sterilized scissors and tweezers, quickly placed in liquid nitrogen, and then transferred to the − 80 °C refrigerator.

### Tissue material and RNA extraction

The total RNA of the sample is extracted by TRIzol reagent (Invitrogen, Carlsbad, CA, USA). The concentration of total RNA was determined by NanoDrop 2000 ultraviolet spectrophotometer (Thermo, USA). The OD 260/280 was required to be 1.8–2.2. Then, the quality of RNAs were examined by Agilent Technologies 2100 Bioanalyze with a minimum RNA integrity number (RIN) of 8.9.

### cDNA library and small RNA preparation and sequencing

Six sequencing libraries were constructed, three libraries (F1, F2, F3) from female groups and other three (M1, M2, M3) from male groups. The samples were prepared by using a mirVana miRNA Isolation Kit (Invitrogen, USA). The libraries were constructed by using TruSeq Stranded mRNA Library Preparation Kit (Illumina, San Diego, CA, USA) according to the manufacturer’s instructions. After passing the quality test of Agilent 2100 Bioanalyzer, the constructed library was sequenced by Illumina HiSeq X Ten sequencer to produce the double-terminal data of 150 bp.

The miRNA library was prepared by using mirVana miRNA Isolation Kit (Invitrogen, USA). The main experimental steps were the ligation of 3′ and 5′ connectors. The small RNAs equipped with connectors were reversed transcript and amplified by PCR. Then 147 nt and 157 nt bands were recovered by RNA gel electrophoresis. The concentration of RNA was detected by Agilent 2100 Bioanalyzer. The platform of small RNA sequencing was Illumina HiSeq 4000. Transcriptome and small RNA sequencing were both completed by OE biotech company (Shanghai).

### Assembly and annotation

A large number of mRNA‐seq raw reads were obtained from male and female gonadal tissues. Remove adaptor, low quality reads and containing ploy-N reads from the raw reads. The clean reads were assembled into expressed sequence tag clusters (contigs). The transcripts were assembled with Trinity (vesion 2.4)^75^. The longest transcript was selected as a unigene based the similarity and length of a sequence analysis. The transcriptome analysis was conducted by OE biotech company (Shanghai, China). The transcript annotations give functional annotations for NR (ftp://ftp.ncbi.nih.gov/blast/db), SwissProt (http://www.uniprot.org/downloads), Clusters of orthologous groups for eukaryotic complete genomes (KOG) (ftp://ftp.ncbi.nih.gov/pub/COG/KOG/kyva), Pfam, GO (Gene Ontology) classification, and KEGG (Kyoto Encyclopedia of Genes and Genomes). The Pfam database was compared with the protein family model by HMMER^76^ software. Other databases were annotated by Diamond^77^ software with a threshold E-value of 10^−5^. Base on the SwissProt annotation, GO classification was performed by the mapping relation between SwissProt and GO term. The unigenes were mapped to the KEGG^78,79^ database to annotate their potential metabolic pathways.

The small RNA sequencing reads were converted into sequence data (also called raw data/reads) by base calling. After filtering out the reads without 3′ adapter and insert tag, reading shorter than 15 nt and longer than 41nt, the clean reads were obtained. The clean reads sequences were compared with Rfam v.10.1 (http://www.sanger.ac.uk/software/Rfam) by blastn. rRNA, scRNA, Cis-reg, snRNA, tRNA and other sequences were annotated. And the transcripts and repetitive sequences were filtered. The filtered sequence was used to identify the known miRNAs by miRBase v.21 database (http://www.mirbase.org/). Unannotated small RNAs were analyzed by mirDeep2^80^. The targets of differentially expressed miRNAs were predicted by using software miRanda^81^, with the parameter as follows: S ≥ 150 ΔG ≤  − 30 kcal/mol and demand strict 5′ seed pairing.

### DEGs and DEMs analysis, GO and KEGG enrichment

Bowtie2 and eXpress were applied to analyze FPKM (fragments per kilobaseper million mapped reads) of each unigene. The counts number of each sample unigene was standardized by using DESeq software. The fold change was calculated. NB (negative binomial distribution test) was used to test the difference significance of reads number. *P* value adjusted with a false-discovery rate (FDR) correction for multiple testing by Benjamini–Hochberg method^82^. Finally, the DEGs were screened according to the |fold change|> 2 and *P* < 0.01. After getting the DEGs, GO and KEGG enrichment analysis were carried out to determine the biological functions or pathways. The number of differential mRNAs included in each GO term was counted. The significance of differential gene enrichment in each GO term was calculated by hypergeometric distribution test. The result of the calculation will return a *P* value of enrichment significance. And the small *P* indicates that the differential unigene is enriched in the GO term. Similarly, KEGG database was used to for analysis DEGs. The hypergeometric distribution test method was used to calculate the significance of differential unigene enrichment in each Pathway. At the same time, the unsupervised hierarchical clustering of DEGs is carried out. And the expression pattern of DEGs among different samples is shown in the form of heat map.

miRNA expression is calculated using TPM (transcript per million), TPM = the number of read compared to each miRNA / the total ratio of samples to the number of read × 10^6^. The miRNAs with *P* value < 0.05 and fold change more than 2 times were selected as differentially expressed miRNAs. Using unsupervised hierarchical clustering analysis, a heat map is constructed based on differentially expressed miRNAs. The heat map (Fig. 1) is drawn by the pheatmap package in R^83^. GO enrichment and KEGG pathway enrichment analysis of different expressed miRNA-target-Gene were respectively performed using R based on the hypergeometric distribution. Figure 3a is drawn by VennDiagram package in R^84^. Figure 3b,c are drawn by ggplot2 R package^85^.

### qRT-PCR of mRNA and miRNAs

We selected 12 genes and 8 miRNAs to verify the sequencing results by qRT-PCR. The primer sequences of mRNA and miRNA are shown in supplementary Table 3 and supplementary Table 4, respectively. For the verification of differentially expressed genes, the kit for RNA reverse transcription is PrimeScript RT reagent Kit (TaKaRa, Japan). The total qRT-PCR reaction of mRNA contained 2 × TB Green Premix Ex Taq II (TaKaRa, Japan) 10 μL, forward and reverse gene-specific primer (10 mM) 0.8 μL, cDNA template 1.6 μL, ddH_2_O 6.8 μL. The condition of the reaction is 95 °C (pre-denaturation) for 15 min, 95 °C (degeneration) for 10 s, and 60 °C (annealing) for 30 s (40 cycles), followed by dissociation curve analysis at 95 °C for 15 s, 60 °C for 1 min and 95 °C for 15 s. EF1α was used as the internal reference gene. The reverse transcription of miRNAs was performed using the Mir-X miRNA First-Strand Synthesis kit (TaKaRa, Japan). The total qRT-PCR reaction of miRNAs contained ddH_2_O 9 μL, TB green advantage premix 12.5 μL, Rox dye 0.5 μL, miRNA specific primer (or U6 forward primer) 0.5 μL, MRQ 3′primer (or U6 reverse primer) 0.5 μL, and cDNA template 2.0 μL. The reaction procedure was set according to the following procedure: 95 °C for 10 s; 95 °C for 5 s, 60 °C for 20 s, 39 cycles; then the PCR temperature was increased from 60 to 95 °C to generate the dissolution curve. U6 snRNA was used as internal reference gene. All qRT-PCR were performed using Bio-Rad CFX-96 (Bio-Rad, USA). The relative expression of mRNAs and miRNAs was calculated by 2^−ΔΔCT^ method. The difference was calculated by SPSS12.0. Statistically significant differences were examined by paired *t* test. A value of *P* < 0.05 was considered to statistically significant.

## Supplementary information

Supplementary Figures 1.

Supplementary Table 1.

Supplementary Table 2.

Supplementary Table 3.

Supplementary Table 4.

## Data Availability

The datasets generated and analyzed during this are available from the corresponding author on reasonable request.
