# Supplementary material for: Integrated mRNA and miRNA expression profile analysis of female and male gonads in Hyriopsis cumingii
Source: Sci Rep. 2021 Jan 12;11:665. doi: 10.1038/s41598-020-80264-7 (PMC7804246; doi:10.1038/s41598-020-80264-7)
Supplement: Supplementary file 1 — Supplementary Figures 1. [file 41598_2020_80264_MOESM1_ESM.docx]

Integrated mRNA and miRNA expression profile analysis of female and male gonads in *Hyriopsis cumingii*

**Ya-Yu Wang ^1,2,3^, Sheng-Hua Duan ^1,2,3^, Gui-Ling Wang ^1,2,3,^*, Jia-Le** **Li ^1,2,3^**

**^1^ Key Laboratory of Freshwater Aquatic Genetic Resources, Ministry of Agriculture and Rural Affairs** **Shanghai Ocean University, Shanghai, 201306;**

**^2^ National Demonstration Center for Experimental Fisheries Science Education, Shanghai, 201306;**

**^3^ Shanghai Engineering Research Center of Aquaculture, Shanghai, 201306;**

*** Corresponding author. 999 Huchenghuan Road, Shanghai Ocean University.**

**E-mail: yhwuwang2008@126.com (G. Wang)**


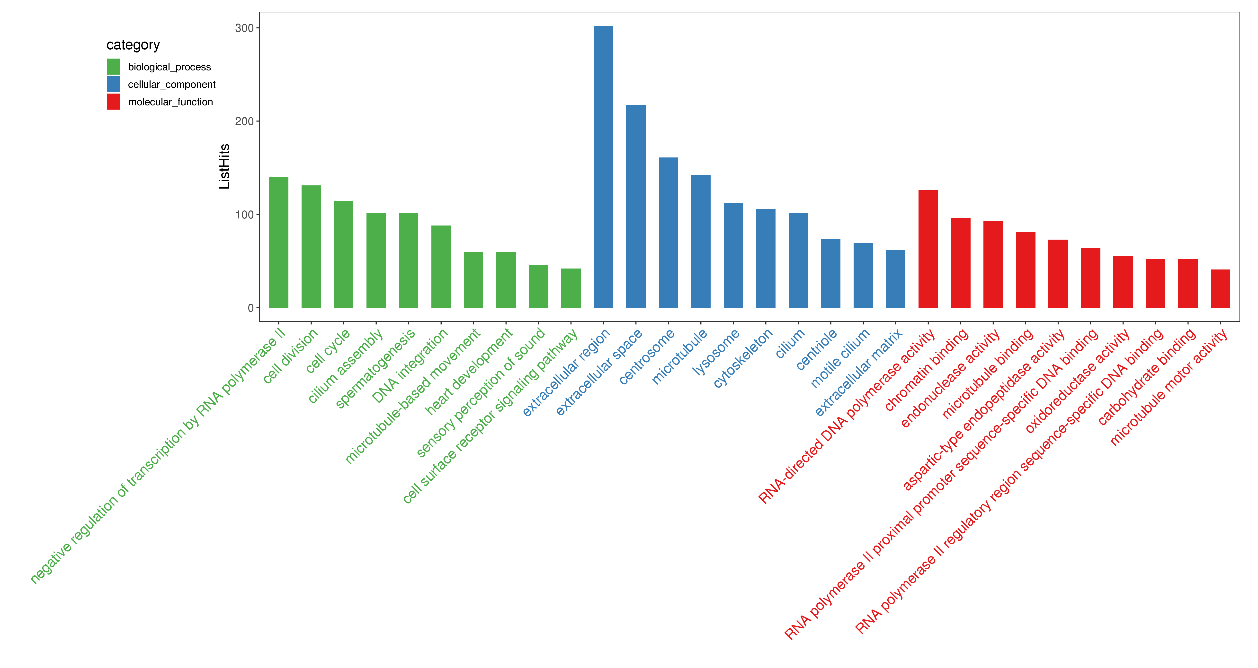


**Supplementary fig 1.** GO enrichment analysis of DEGs between between Group_F vs Group_M in *H.cumingii*. The x-axis indicates term names and y-axis indicates the number of differentially expressed genes in each term.


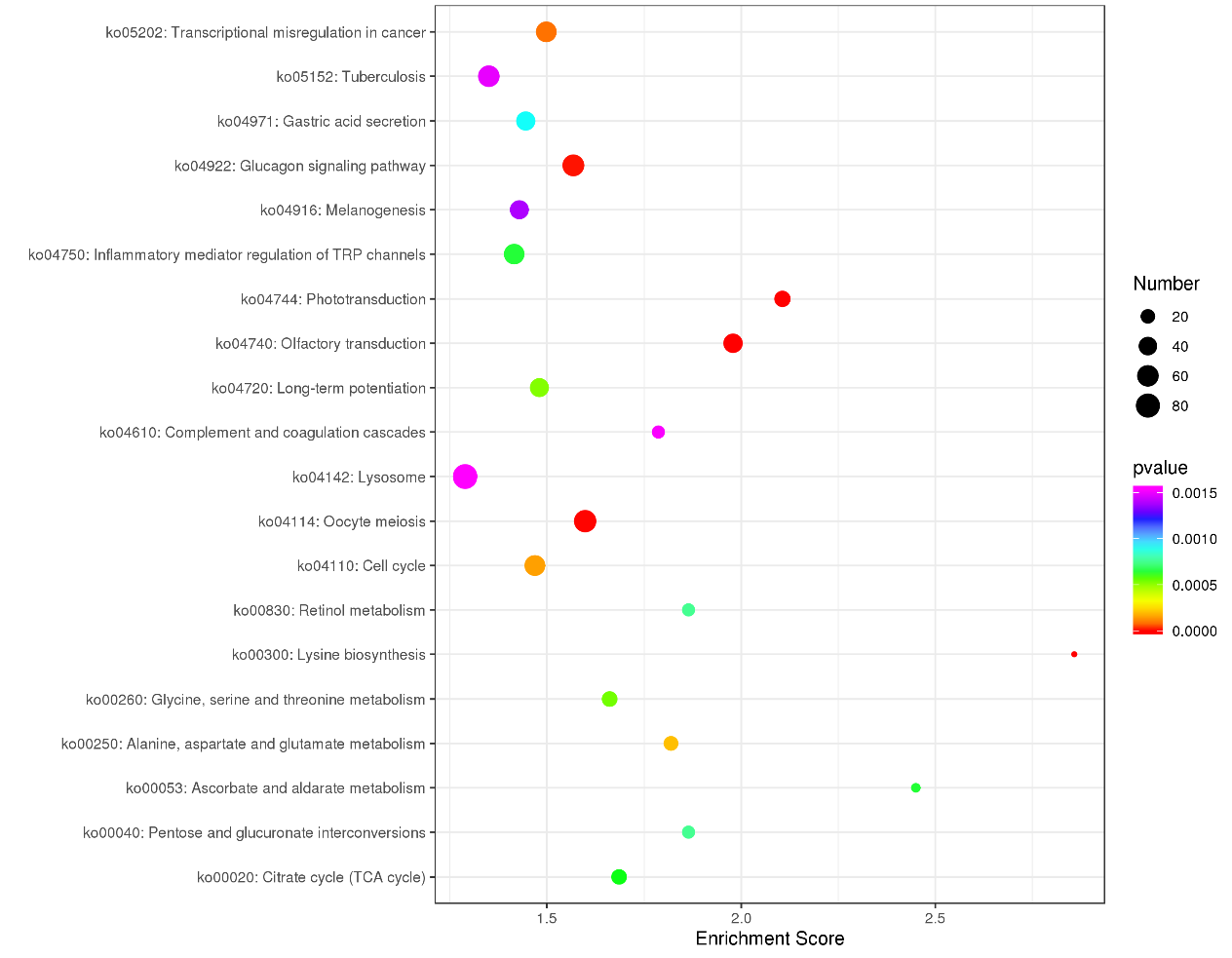


**Supplementary fig 2.** KEGG enrichment analysis of DEGs between Group_F vs Group_M in *H.cumingii*. The color of the bubble changes from purple-blue-green-red, the smaller the value of its enrichment p-value, the greater the saliency.


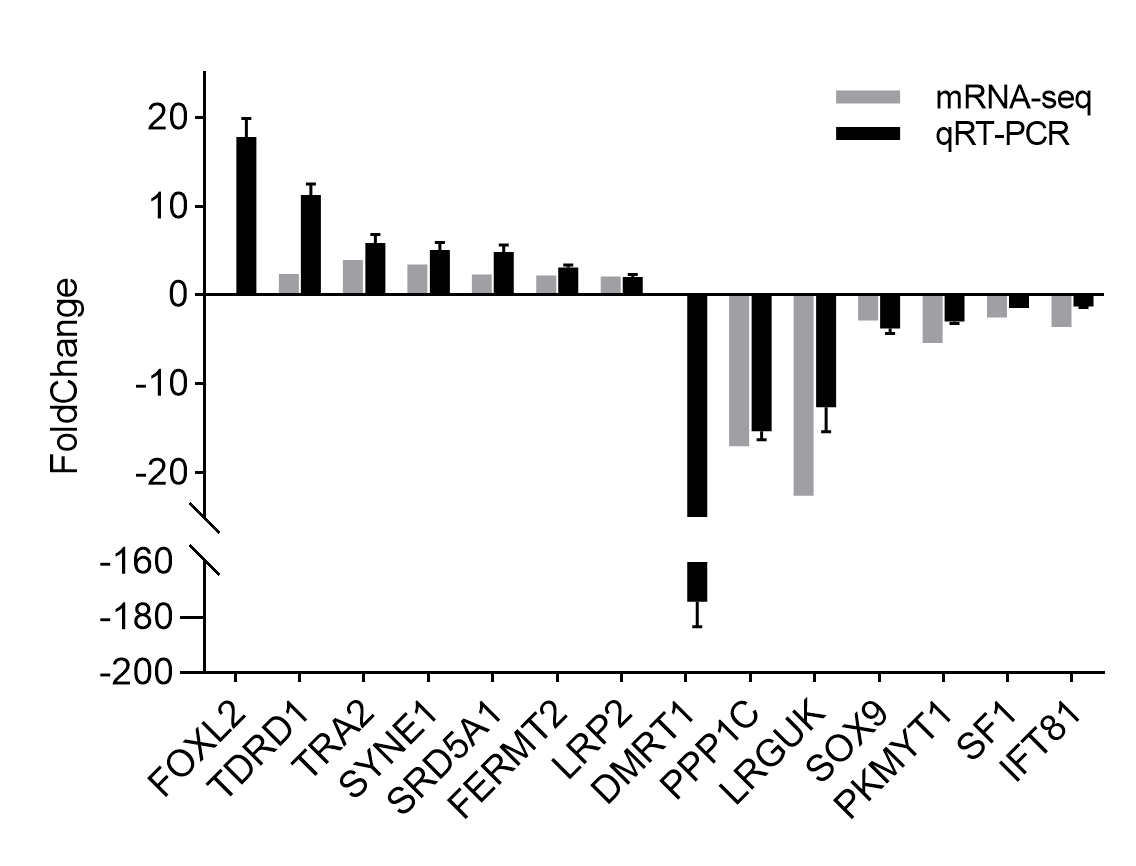


**Supplementary fig 3.** The relative expression of different mRNAs revealed by real-time quantitative PCR. Data are shown as mean ± SD (standard deviation) of tissues from three separate individuals.


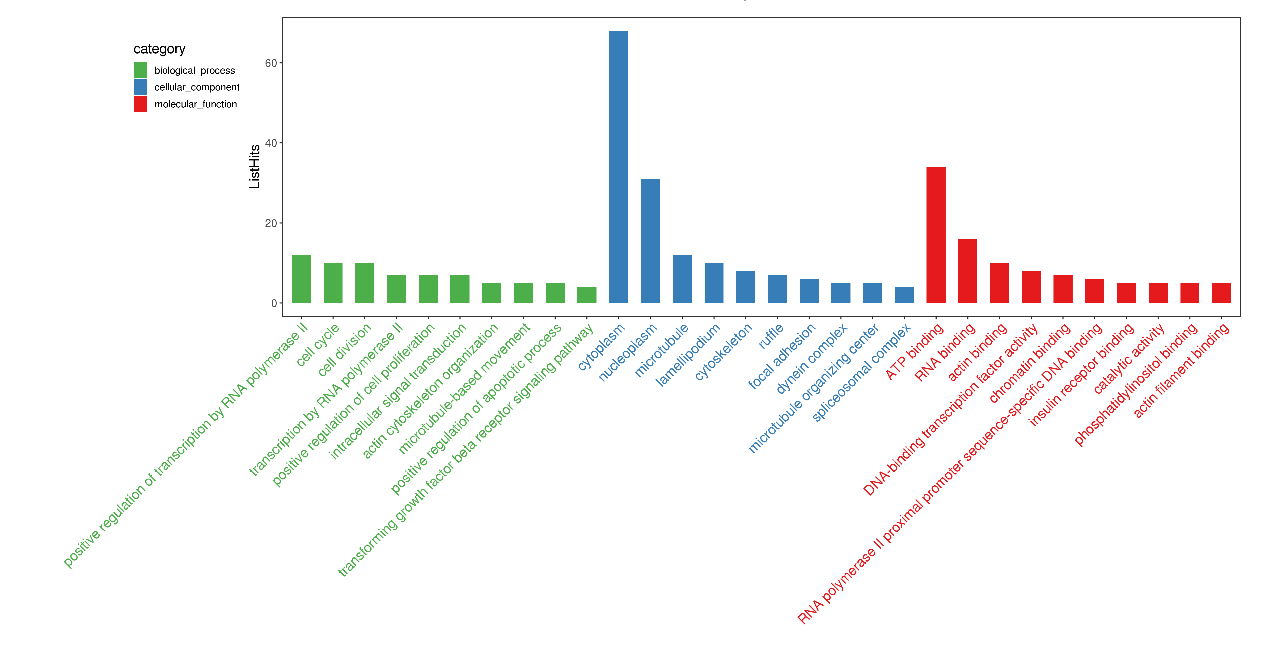


**Supplementary fig 4.** GO enrichment analysis of target genes of 32 DEMs in *H.cumingii*. The x-axis indicates term names and y-axis indicates the number of differentially expressed genes in each term.


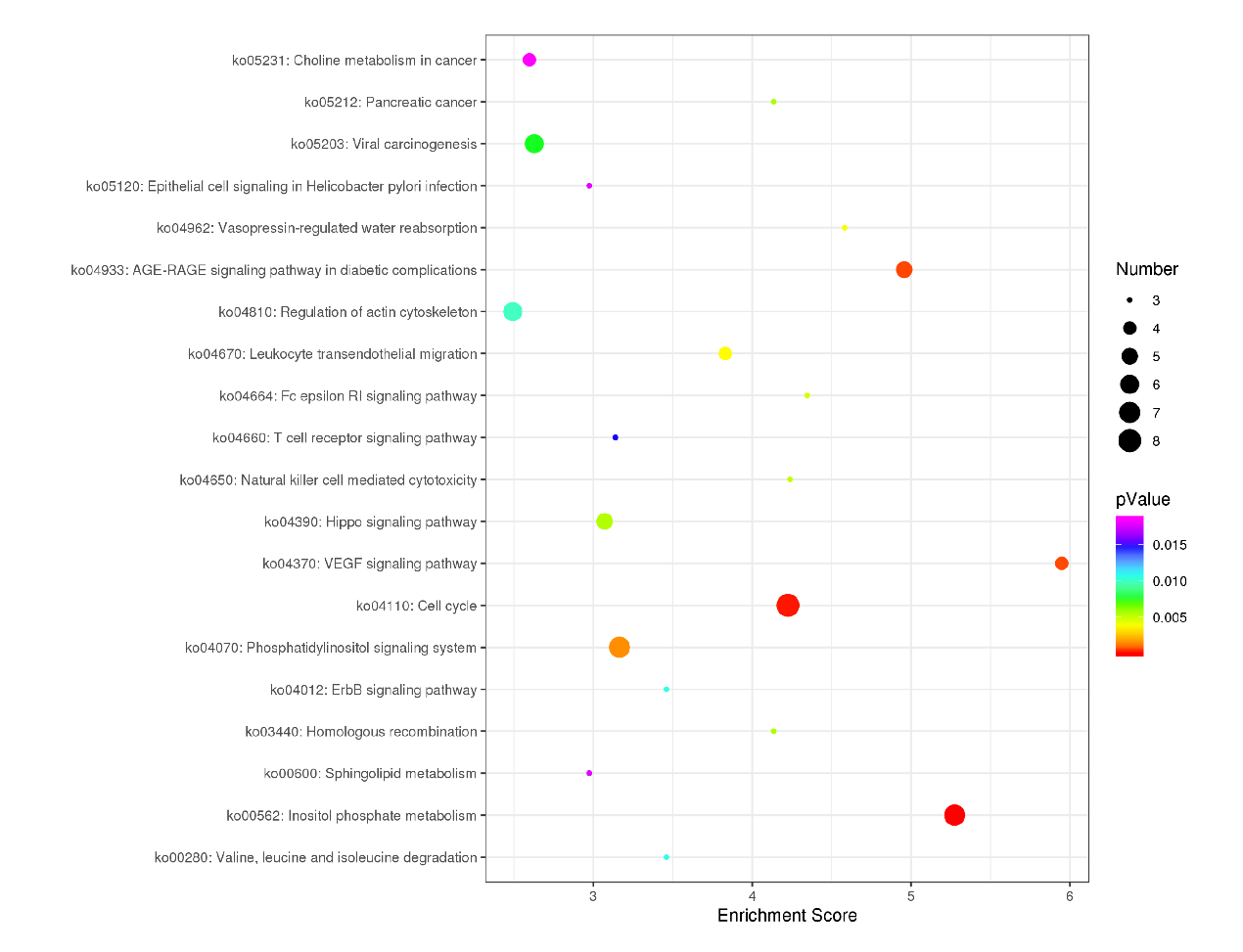


**Supplementary fig 5.** KEGG enrichment analysis of target genes of 32 DEMs in *H.cumingii*. The color of the bubble changes from purple-blue-green-red, the smaller the value of its enrichment p-value, the greater the saliency.
